# ADCC-activating antibodies correlate with decreased risk of congenital human cytomegalovirus transmission

**DOI:** 10.1172/jci.insight.167768

**Published:** 2023-07-10

**Authors:** Eleanor C. Semmes, Itzayana G. Miller, Nicole Rodgers, Caroline T. Phan, Jillian H. Hurst, Kyle M. Walsh, Richard J. Stanton, Justin Pollara, Sallie R. Permar

**Affiliations:** 1 Medical Scientist Training Program, Department of Molecular Genetics and Microbiology, and; 2 Duke Human Vaccine Institute, Duke University, Durham, North Carolina, USA.; 3 Department of Pediatrics, Weill Cornell Medicine, New York City, New York, USA.; 4 Department of Surgery, Duke University School of Medicine, Durham, North Carolina, USA.; 5 Department of Pediatrics, Duke University, Durham, North Carolina, USA.; 6 Department of Neurosurgery, Duke University, Durham, North Carolina, USA.; 7 Division of Infection and Immunology, School of Medicine, Cardiff University, Cardiff, United Kingdom.

**Keywords:** Immunology, Infectious disease, Adaptive immunity, Immunoglobulins, NK cells

## Abstract

Human cytomegalovirus (HCMV) is the most common vertically transmitted infection worldwide, yet there are no vaccines or therapeutics to prevent congenital HCMV (cCMV) infection. Emerging evidence indicates that antibody Fc effector functions may be a previously underappreciated component of maternal immunity against HCMV. We recently reported that antibody-dependent cellular phagocytosis (ADCP) and IgG activation of FcγRI/FcγRII were associated with protection against cCMV transmission, leading us to hypothesize that additional Fc-mediated antibody functions may be important. In this same cohort of HCMV-transmitting (*n* = 41) and nontransmitting (*n* = 40) mother-infant dyads, we report that higher maternal sera antibody–dependent cellular cytotoxicity (ADCC) activation is also associated with lower risk of cCMV transmission. We investigated the relationship between ADCC and IgG responses against 9 viral antigens and found that ADCC activation correlated most strongly with sera IgG binding to the HCMV immunoevasin protein UL16. Moreover, we determined that higher UL16-specific IgG binding and FcγRIII/CD16 engagement were associated with the greatest risk reduction in cCMV transmission. Our findings indicate that ADCC-activating antibodies against targets such as UL16 may represent an important protective maternal immune response against cCMV infection that can guide future HCMV correlates studies and vaccine or antibody-based therapeutic development.

## Graphical abstract

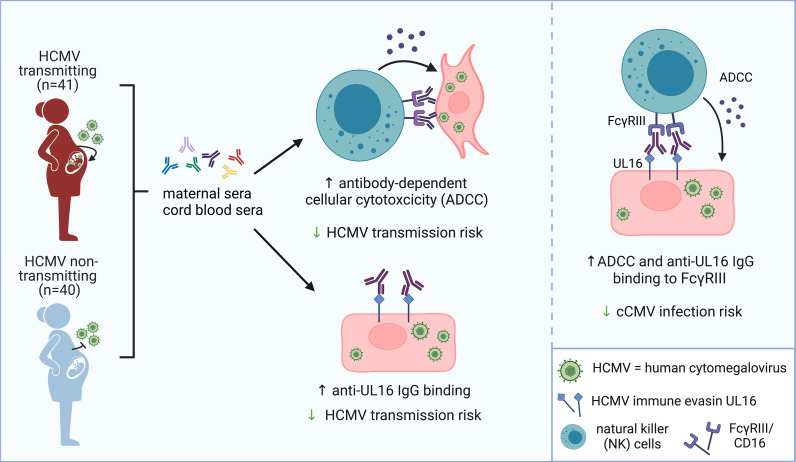

## Introduction

Human cytomegalovirus (HCMV) is the most common vertically transmitted infection worldwide and has been associated with stillbirth, neurodevelopmental impairment, sensorineural hearing loss, and childhood leukemia (1, 2). Over 80% of reproductive-age women worldwide are HCMV seropositive, and congenital transmission can occur following primary or nonprimary HCMV infection, which may include reinfection with a new strain or reactivation from viral latency (3). Despite these disease risks and the ubiquity of congenital HCMV (cCMV) infection, we lack effective therapeutics and vaccines to prevent HCMV transmission. Neutralizing antibodies against HCMV entry envelope glycoproteins (e.g., glycoprotein B [gB] and pentamer complex) and T cell responses have been the main targets in vaccine development to date, but these vaccines have achieved only limited to moderate efficacy (4). Moreover, several studies have found that maternal neutralizing antibody titers do not correlate with reduced risk of cCMV infection (5, 6). We also recently reported that neutralizing antibody titers against multiple HCMV strains and cell types were higher magnitude in HCMV-transmitting pregnancies and were not associated with protection (7). Maternal treatment with HCMV hyperimmunoglobulin (HCMV-HIG), a pooled polyclonal preparation of IgG from HCMV-seropositive donors, following primary infection during pregnancy also failed to prevent congenital transmission in 2 randomized clinical trials (8, 9). Thus, an improved understanding of the maternal antibody responses that protect against cCMV transmission is urgently needed to guide the development of vaccines and immunotherapeutics (10, 11).

Antibodies can mediate polyfunctional responses including neutralization through the Fab region that binds antigen and nonneutralizing functions through the constant Fc region that binds Fc receptors (FcRs) on innate immune cells. Emerging evidence indicates that anti-viral IgG effector functions mediated by interactions between the IgG Fc region and FcγRs are a previously underappreciated component of anti-HCMV immunity (12, 13). In our recent study reporting that maternal neutralizing antibody titers were not correlated with protection, we found that greater nonneutralizing Fc-mediated antibody responses were associated with reduced risk of transmission (7). Specifically, higher maternal sera anti-HCMV IgG engagement of FcγRI/FcγRIIa and activation of antibody-dependent cellular phagocytosis (ADCP) were associated with decreased risk of vertical transmission. Therefore, we posited that additional antibody Fc effector functions may be important.

Antibody-dependent cellular cytotoxicity (ADCC) is an antiviral Fc effector function that has been underexplored in cCMV infection to date. NK cells can eliminate virally infected cells via ADCC, which is mediated by FcγRIII/CD16 expressed on the NK cell surface, or through direct cytotoxic killing. HCMV has evolved many mechanisms to evade antibody-dependent and -independent NK cell killing by encoding “immunoevasins” that interfere with the host immune response (14, 15). Specific immune evasion strategies employed by HCMV include viral FcγR decoys that sequester IgG to prevent FcγRIII activation (16, 17) and viral proteins that modulate NK cell cytotoxicity (15, 18). Nevertheless, NK cell–mediated ADCC can inhibit cell-to-cell spread of HCMV in multiple cell types (19, 20), even with strains that express these viral immunoevasins (21). Paradoxically, some NK cell immunoevasins (e.g., viral FcγR gp34, UL16, and UL141) have even been identified as targets of ADCC-activating IgG (21). Despite evidence that ADCC mediated by certain IgG specificities can overcome viral immune evasion, whether ADCC or anti-immunoevasion antibodies help limit HCMV transmission in utero is unknown.

In this study, we hypothesized that greater ADCC and FcγRIII/CD16 engagement by maternal antibodies would be correlated with reduced risk of cCMV transmission. To investigate this hypothesis, we quantified ADCC and FcγRIII activation in maternal and cord blood sera from HCMV transmitting and nontransmitting pregnancies (7). We then explored whether HCMV-specific IgG against different HCMV antigens (e.g., envelope glycoproteins, tegument proteins, and immunoevasins) might contribute to protective ADCC responses. Our work suggests that Fc-mediated immunity and IgG against noncanonical HCMV antigens may be important for preventing HCMV transmission in utero. These findings can guide future work to define the role of ADCC-activating antibodies in antiviral control and support mounting evidence that Fc-mediated antibody functions should be considered as immunologic targets in HCMV correlates and vaccine studies.

## Results

### Overview of mother-infant cohort.

To determine whether ADCC-mediating antibodies were correlated with protection against cCMV infection, we compared antibody responses in maternal and cord blood sera from HCMV seropositive transmitting (*n* = 41) and nontransmitting (*n* = 40) mother-infant dyads that we previously identified as donors to the Carolinas Cord Blood Bank (CCBB), a large US-based public cord blood bank (Supplemental Figure 1; supplemental material available online with this article; https://doi.org/10.1172/jci.insight.167768DS1) (7). Cases of cCMV infection were identified based on the detection of HCMV DNAemia in the cord blood plasma and dyads were matched on infant sex, race, maternal age, and delivery year. Cord blood donors were screened for clinical signs of (a) neonatal sepsis, (b) congenital infection (petechial rash, hepatosplenomegaly, thrombocytopenia), and (c) congenital abnormalities at birth, and only term, healthy, uncomplicated births were included. Demographic and clinical characteristics were comparable between groups (Table 1) (7), but HCMV serologies differed between transmitting and nontransmitting dyads. Median HCMV IgG relative avidity index (RAI) scores were lower in transmitting (median = 67.7%) versus nontransmitting (median = 74.8%) pregnancies, and 19.5% of transmitting cases were classified as low/intermediate avidity (<60%), whereas 2.5% of nontransmitting pregnancies had low/intermediate RAI scores (Table 1). Additionally, 26.8% of transmitting pregnancies had HCMV-specific IgM in maternal sera versus only 5% of nontransmitting pregnancies (Table 1). While it is not possible to define primary infection, reinfection, or reactivation based on HCMV serologies at 1 time point (22, 23), these data imply that the timing of maternal HCMV exposure may have differed between groups.

### HCMV-specific ADCC and FcγRIII/CD16 activating antibodies are higher in nontransmitting versus transmitting pregnancies.

**Figure 1:**
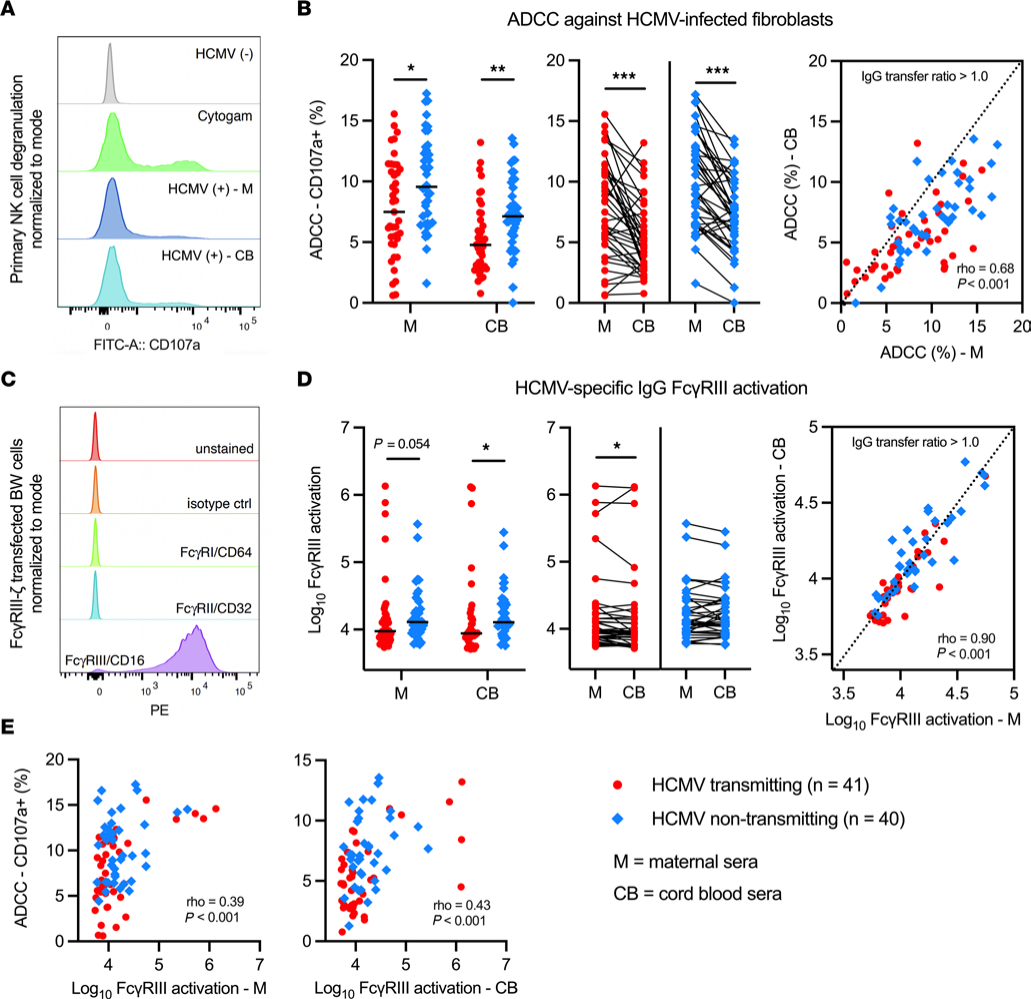
HCMV-specific ADCC and FcγRIII/CD16 activating antibodies in HCMV transmitting versus nontransmitting mother-infant dyads. HCMV-specific ADCC and FcγRIII IgG activation was measured using maternal (M) and cord blood (CB) sera from HCMV transmitting (red circles, *n* = 41) and nontransmitting (blue diamonds, *n* = 40) mother-infant dyads. Antibody responses were compared between and within dyads. (**A**) NK cell degranulation (% CD107a^+^ NK cells; gating strategy in Supplemental Figure 2) was quantified as a read-out of ADCC using a flow-based assay. Primary NK cells were isolated from PBMCs by negative selection with magnetic beads prior to coincubation with HCMV-infected and mock-infected cells. Cytogam (light green), HCMV seropositive (light and dark blue), and HCMV seronegative (gray) sera samples were included as controls. (**B**) HCMV-specific antibody ADCC responses in transmitting and nontransmitting dyads. (**C**) Flow cytometry of FcγR-CD3ζ BW cells showing unstained (red), isotype control (orange), anti-FcγRI/CD64 (light green), anti-FcγRII/CD32 (blue), and anti-FcγRIII/CD16 (purple) PE–conjugated antibody staining. (**D**) Anti–HCMV IgG FcγRIII activation in transmitting and nontransmitting dyads. (**E**) Scatterplots showing Spearman correlations between HCMV-specific ADCC and FcγRIII IgG activation. IgG transfer ratio equals paired cord blood/maternal sera responses. Horizontal black bars denote median. (**A** and **D**) FDR-corrected *P* values for Mann-Whitney *U* test (left) or Wilcoxon signed-rank test (right). *P < 0.05, **P < 0.01, ***P < 0.001.

Using NK cell degranulation (i.e., percentage of CD107a^+^ NK cells) against HCMV-infected fibroblasts to quantify ADCC (Supplemental Figure 2, A and B, and Figure 1A), we found that antibodies in sera from nontransmitting dyads mediated significantly greater ADCC compared with transmitting dyads (Figure 1B and Supplemental Tables 1 and 2). ADCC responses in paired cord blood and maternal sera were strongly correlated, yet NK cell degranulation was significantly lower in cord blood versus maternal sera (Figure 1B). Few dyads had placental IgG transfer ratios > 1.0 or 100% (Figure 1B), suggesting that the transfer of ADCC-activating IgG into fetal circulation was low. In our univariate regression analysis, higher ADCC activation in maternal (OR = 0.86, *P* = 0.016) and cord blood sera (OR = 0.79, *P* = 0.005) was associated with lower risk of transmission (Tables 2 and 3). Moreover, ADCC-activating antibodies were associated with a greater magnitude reduction in transmission risk compared with our previously identified immune correlate of ADCP-activating antibodies (OR = 0.94, *P* = 0.008) (7) (Tables 2 and 3). To assess if differences in maternal HCMV exposure may be confounding these results, we compared ADCC in dyads stratified by RAI score or HCMV-specific IgM status and found that ADCC activation was lower in dyads with low/intermediate (median = 5.1%) versus high (median = 9.5%, *P* = 0.001) RAI scores (Supplemental Tables 3 and 4). Nevertheless, ADCC remained significantly associated with protection in cord blood sera (OR = 0.81, *P* = 0.017) with a trend toward significance in maternal sera (OR = 0.89, *P* = 0.065) in a sensitivity analysis excluding dyads with low/intermediate RAI scores (Supplemental Table 5).

Since ADCC is primarily mediated by FcγRIII/CD16 on NK cells binding to the IgG Fc region, we hypothesized that anti-HCMV IgG in nontransmitting dyads may have enhanced FcγRIII engagement compared with transmitting dyads. To explore this hypothesis, we quantified anti-HCMV IgG activation of FcγRIII using reporter cells expressing chimeric human FcγRIII fused to a mouse CD3ζ signaling domain (Figure 1C). Nontransmitting dyads had significantly higher HCMV-specific IgG FcγRIII activation in cord blood (*P* = 0.008) with a trend toward significance in maternal sera (*P* = 0.054) (Figure 1D and Supplemental Tables 1 and 2). FcγRIII activation was lower in cord blood (median = 8,791 μg/mL) versus maternal sera (median = 9,481 μg/mL, *P* = 0.032) in transmitting but not nontransmitting dyads (Figure 1D), suggesting that infected infants may receive less FcγRIII-activating IgG via placental transfer. HCMV-specific IgG FcγRIII activation correlated with ADCC (Figure 1E) but was not independently associated with transmission risk in our univariate analysis (Tables 2 and 3). Nevertheless, our interaction analysis demonstrated that the association between ADCC and reduced risk of cCMV transmission was stronger in dyads with greater FcyRIII activation (Tables 2 and 3), indicating that higher magnitude FcyRIII engagement contributed to protective ADCC responses.

### ADCC and FcγRIII/CD16 activation correlate with anti-UL16 and anti-UL141 IgG responses.

**Figure 2:**
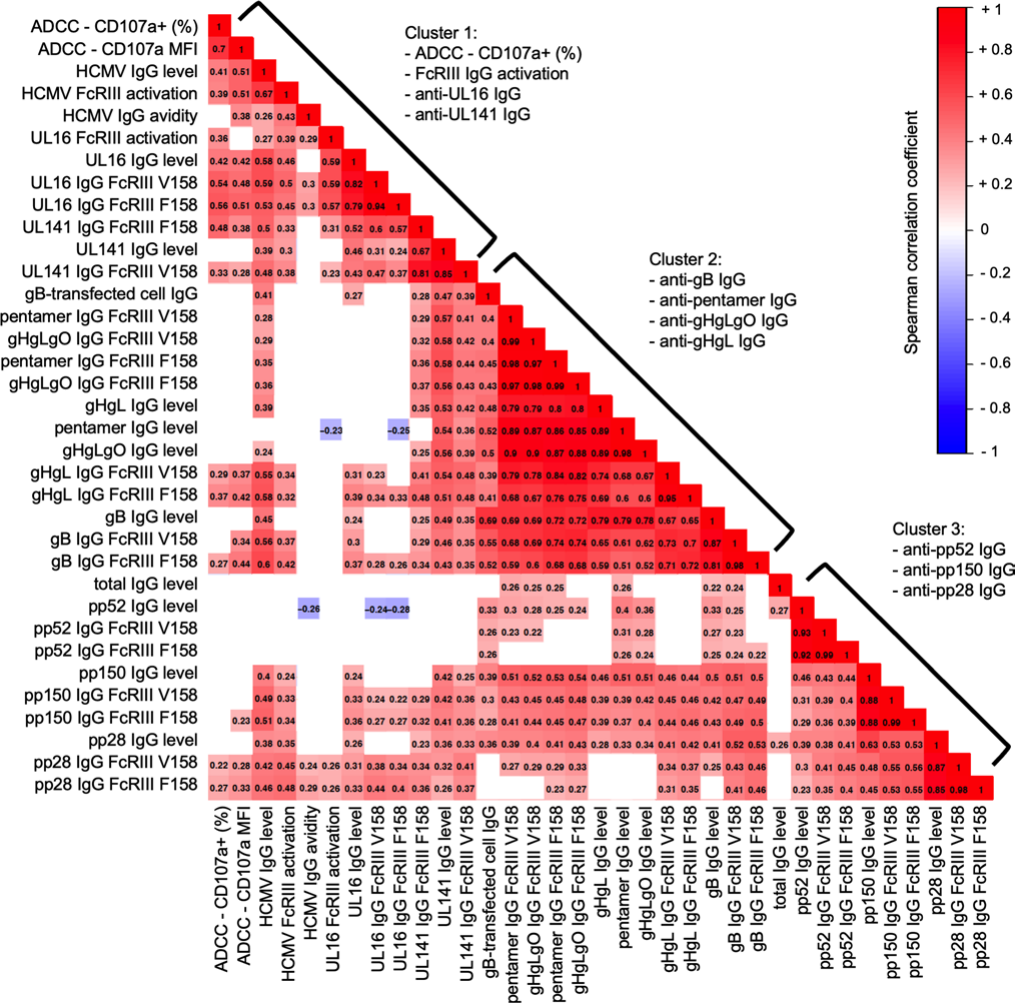
HCMV-specific NK cell ADCC and FcγRIII/CD16 activating antibodies cluster with anti-UL16 and anti-UL141 IgG responses. Hierarchical clustering was performed on Spearman correlation coefficients to group strongly correlated immune variables. Matrix of maternal sera antibody responses showing Spearman correlation coefficients from –1.0 (blue) to +1.0 (red). Nonsignificant correlations (*P* > 0.05) shown in white. MFI, mean florescent intensity; level, total antigen-specific IgG binding measured by a binding antibody multiplex assay; gB-transfected cell IgG, IgG binding to cell-associated gB as measured in ref. 7.

Having identified that ADCC-activating antibodies were associated with protection against cCMV, we sought to define which antibodies may be contributing to this response. We hypothesized that IgG against viral immunoevasins (e.g., UL16- and UL141-specific IgG) may activate greater ADCC in nontransmitting dyads based on recent work by Vlahava et al. (21). We also examined IgG against HCMV envelope glycoproteins (e.g., gB, gH/gL, gH/gL/gO, pentamer complex) and tegument proteins (e.g., pp52, pp28, pp150) since these antigens are targeted by robust host IgG responses, yet whether they activate ADCC has been poorly characterized. Using binding antibody multiplex assays (BAMAs), we quantified IgG levels and FcγRIII engagement for 9 HCMV antigens. Since FcγRIII binding is influenced by IgG Fc region characteristics as well as FcγRIII polymorphisms, we included binding to both the high-affinity (V158) and low-affinity (F158) FcγRIII variants. Hierarchical clustering identified 3 distinct groups of antibody responses (Figure 2). HCMV-specific ADCC (i.e., percentage of CD107a^+^ NK cells), FcγRIII activation, anti-UL16, and anti-UL141 IgG responses correlated together in cluster 1, whereas cluster 2 consisted of IgG targeting HCMV envelope glycoproteins, and cluster 3 comprised IgG responses against HCMV tegument proteins. Of the 9 antigen specificities tested, only total anti-UL16 IgG binding levels were significantly correlated with ADCC (ρ = 0.42 *P* < 0.0001; Figure 2), and anti-UL16 IgG binding to FcγRIII V158/F158 correlated most strongly with ADCC/FcγRIII activation (Figure 2). Anti-UL141 IgG binding to FcγRIII V158/F158 was more modestly correlated with ADCC/FcγRIII activation (Figure 2). Despite clustering separately, some anti-gB, anti-gH/gL, anti-pp28, and anti-pp150 IgG FcγRIII binding responses were modestly correlated with ADCC and/or FcγRIII activation (Figure 2). These weaker correlations suggest that IgG against some HCMV envelope glycoproteins or tegument proteins may help mediate ADCC but likely to a lesser degree than UL16- and UL141-specific IgG.

### Magnitude and quality of anti-HCMV IgG binding to FcγRIII differs in nontransmitting and transmitting dyads.

**Figure 3:**
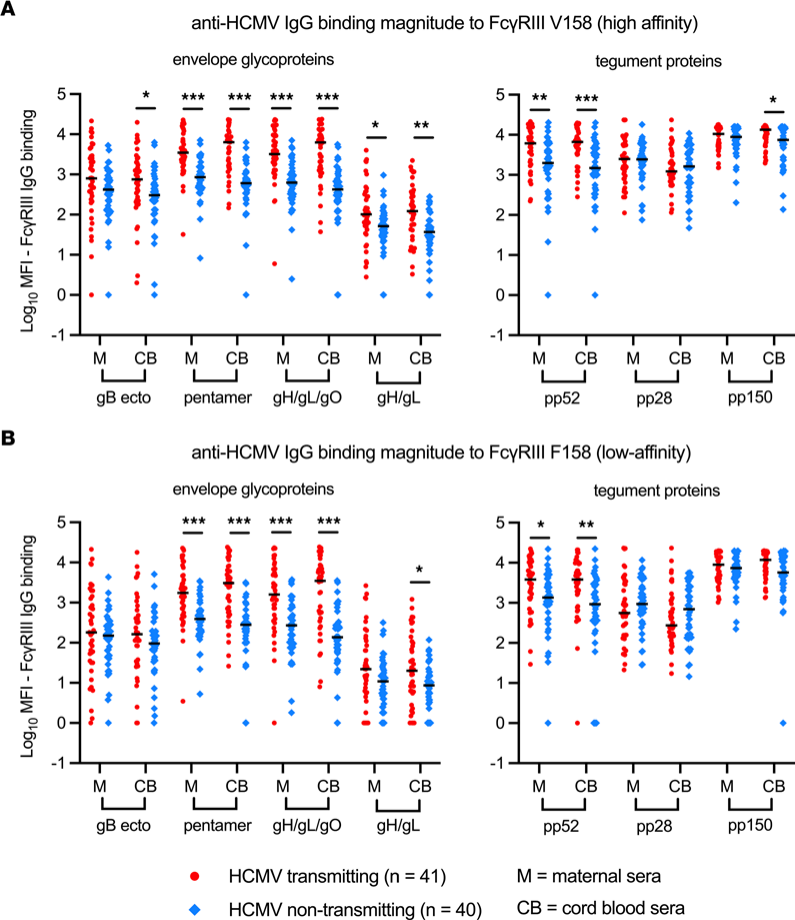
HCMV antigen–specific IgG binding magnitude to FcγRIII/CD16 in transmitting versus nontransmitting dyads. HCMV antigen-specific IgG binding to FcγRIII in maternal (M) and cord blood (CB) sera was measured using a binding antibody multiplex assay with a biotinylated FcγR and streptavidin-PE detection antibody. HCMV antigen–specific IgG binding to FcγRIII was compared between transmitting (red circles, *n* = 41) and nontransmitting (blue diamonds, *n* = 40) mother-infant dyads. (**A**) HCMV antigen-specific IgG binding to FcγRIII high-affinity V158 variant. (**B**) HCMV antigen-specific IgG binding to FcγRIII low-affinity F158 variant. Horizontal black bars denote median. (**A** and **B**) FDR-corrected *P* values reported for Mann-Whitney *U* test. *P < 0.05, **P < 0.01, ***P < 0.001.

**Figure 4:**
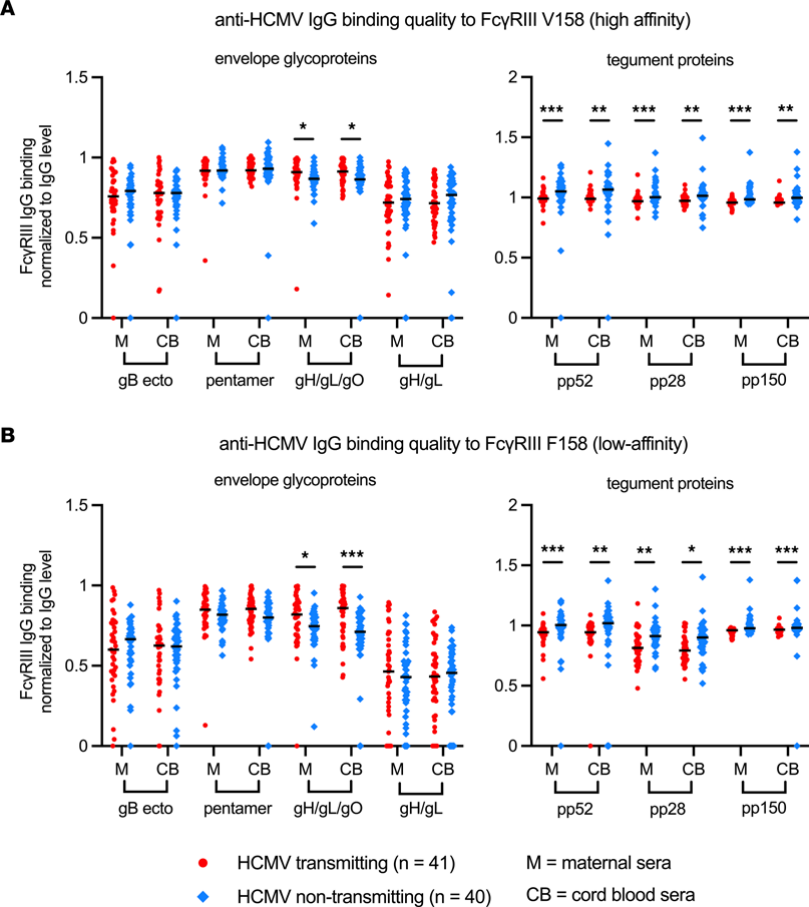
HCMV antigen-specific IgG binding quality to FcγRIII/CD16 in transmitting versus nontransmitting dyads. HCMV antigen-specific IgG binding to FcγRIII in maternal (M) and cord blood (CB) sera was measured using a binding antibody multiplex assay with a biotinylated FcγR and streptavidin-PE detection antibody. Antigen-specific IgG binding to FcγRIII was normalized to total antigen-specific IgG binding (i.e., total antigen-specific IgG level) as a ratio and compared between transmitting (red circles, *n* = 41) and nontransmitting (blue diamonds, *n* = 40) mother-infant dyads. (**A**) Normalized HCMV antigen-specific IgG binding to FcγRIII high-affinity V158 variant. (**B**) Normalized HCMV antigen-specific IgG binding to FcγRIII low-affinity F158 variant. Horizontal black bars denote median. (**A** and **B**) FDR-corrected *P* values reported for Mann-Whitney *U* test. *P < 0.05, **P < 0.01, ***P < 0.001.

We previously reported that anti-HCMV IgG engagement of FcγRI/FcγRIIa was enhanced in nontransmitting dyads despite having lower-magnitude anti-HCMV IgG levels (7). In this follow-up study, we observed a similar phenomenon with FcγRIII. Magnitude of anti-envelope glycoproteins or anti-tegument IgG binding to FcγRIII was higher in transmitting versus nontransmitting dyads (Figure 3, A and B); however, magnitude of IgG binding to FcγRIII incorporates both the strength of Fab binding to the antigen and the Fc-FcγRIII interaction. Since antigen-specific IgG levels differed between groups (Supplemental Tables 1 and 2), we normalized FcγRIII binding to total antigen-specific IgG levels to directly compare the quality of the Fc-FcγRIII interaction. After normalization, we found that the Fc region of anti-tegument IgG from nontransmitters had better binding to both high- and low-affinity FcγRIII (Figure 4, A and B).

### Anti-UL16, but not anti-UL141, IgG responses are associated with reduced risk of HCMV transmission in utero.

**Figure 5:**
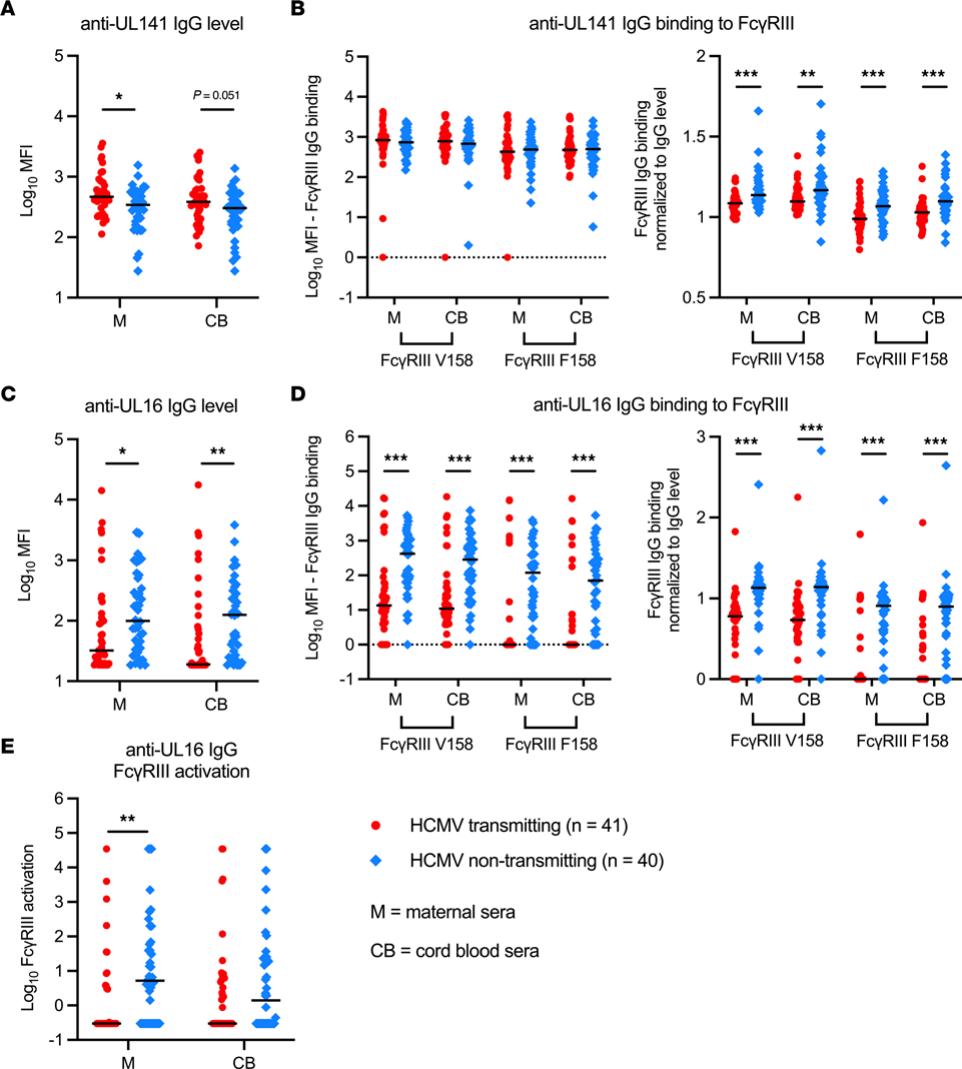
Anti-UL141 and anti-UL16 IgG binding in HCMV transmitting versus nontransmitting mother-infant dyads. Anti-UL141 and anti-UL16 IgG binding was measured with a binding antibody multiplex assay using maternal (M) and cord blood (CB) sera from HCMV transmitting (red circles, *n* = 41) and nontransmitting (blue diamonds, *n* = 40) mother-infant dyads. (**A**) Total anti-UL141 IgG binding (i.e., level). (**B**) Anti-UL141 IgG binding to FcγRIII high-affinity V158 and low-affinity F158 variants before and after normalization for total anti-UL141 IgG level. (**C**) Total anti-UL16 IgG binding (i.e., level). (**D**) Anti-UL16 IgG binding to FcγRIII high-affinity V158 and low-affinity F158 variants before and after normalization for total anti-UL16 IgG level. (**E**) Anti–UL16 IgG FcγRIII activation measured via FcγR-CD3ζ BW cell activation assay. (**A–E**) FDR-corrected *P* values reported for Mann-Whitney *U* test. *P < 0.05, **P < 0.01, ***P < 0.001.

Next, we compared anti-UL141 and anti-UL16 IgG responses in transmitting versus nontransmitting dyads. Although modestly correlated with ADCC, anti-UL141 IgG levels were slightly higher in transmitting pregnancies (Figure 5A and Supplemental Tables 1 and 2) and UL141-specific IgG transfer was low (Supplemental Figure 3A). While the magnitude of anti-UL141 IgG binding to FcγRIII was similar between groups, normalized anti-UL141 IgG binding to FcγRIII V158/F158 was significantly higher in nontransmitters (Figure 5B), suggesting better-quality Fc-FcγRIII engagement. Nevertheless, anti-UL141 IgG binding was not associated with protection against transmission in our univariate regression analysis (Tables 2 and 3). In contrast, anti-UL16 IgG levels were 3-fold higher in maternal (99.8 versus 32.3 MFI, *P* = 0.019) and 6-fold higher in cord blood (125.4 versus 19.0 MFI, *P* = 0.002) sera from nontransmitting compared with transmitting pregnancies (Figure 5C and Supplemental Tables 1 and 2). Anti-UL16 IgG levels were lower in cord blood versus maternal sera within transmitting dyads, whereas anti-UL16 IgG levels were higher in cord blood versus maternal sera in nontransmitting dyads (Supplemental Figure 3B). These data indicate that transplacental transfer of UL16-specific IgG was increased in nontransmitting versus transmitting pregnancies. Anti-UL16 IgG in maternal sera of nontransmitting dyads also had 30- to 100-fold higher magnitude binding to FcγRIII V158 (420.6 versus 13.5 MFI, *P* < 0.0001) and F158 (119.4 versus 1.0 MFI, *P* < 0.0001) and better-quality Fc-FcγRIII binding after normalization for anti-UL16 IgG levels (Figure 5D). When quantified with our chimeric human FcγRIII reporter cells, anti-UL16 IgG in maternal sera from nontransmitters also had higher functional activation of FcγRIII (Figure 5E). In our univariate regression analysis, anti-UL16 IgG binding to and activation of FcγRIII were significantly associated with reduced risk of cCMV transmission (Tables 2 and 3). When comparing anti-UL16 IgG responses in dyads stratified by RAI score and HCMV-specific IgM status, anti-UL16 IgG binding was lower in dyads with low/intermediate RAI scores or HCMV-specific IgM (Supplemental Tables 3 and 4). Nevertheless, anti-UL16 IgG binding to and activation of FcγRIII remained associated with reduced transmission risk in a sensitivity analysis excluding these dyads (Supplemental Tables 5 and 6).

### Anti-UL16 IgG activates NK cell ADCC in HCMV nontransmitting pregnancies.

**Figure 6:**
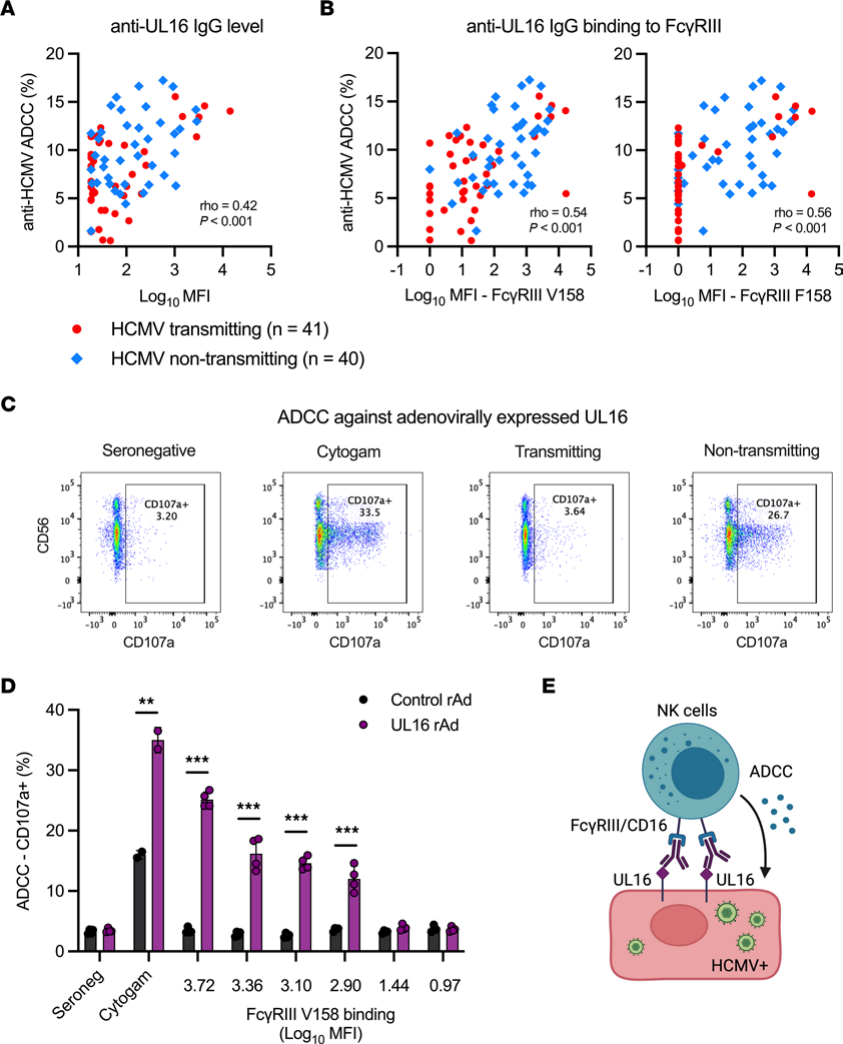
Anti-UL16 IgG activates NK cell ADCC in HCMV nontransmitting pregnancies. (**A**–**D**) ADCC activation was quantified as NK cell degranulation (% CD107a^+^ NK cells; gating strategy in Supplemental Figure 2) against fibroblasts infected with HCMV (**A** and **B**) or transduced with a recombinant adenovirus (rAd) expressing UL16 or a control rAd (**C** and **D**). Anti-UL16 IgG binding was measured with a binding antibody multiple assay. (**A** and **B**) Scatterplots of maternal sera responses from transmitting (red circles, *n* = 41) and nontransmitting (blue diamonds, *n* = 40) dyads. (**A**) Spearman correlation between anti-HCMV ADCC and anti-UL16 IgG levels. (**B**) Spearman correlation between anti-HCMV ADCC and anti-UL16 IgG binding to FcγRIII high-affinity V158 and low-affinity F158 variants. (**C** and **D**) NK cell degranulation against UL16rRAd (purple) and control rAd (black) using Cytogam (positive control), seronegative sera (negative control), and a limited subset of maternal sera samples (*n* = 6) selected based on sera volume availability. Dots represent biological replicates tested at 1:10 and 1:50 sera dilution. Serum samples with a range of representative anti–UL16 IgG FcγRIII binding responses were included. (**E**) Hypothetical model demonstrating proposed role for anti-UL16 IgG binding to FcγRIII in mediating NK cell ADCC against HCMV-infected cells. (**E**) FDR-corrected *P* values reported for Mann-Whitney *U* test. **P < 0.01, ***P < 0.001.

Finally, we investigated the relationship between anti-UL16 IgG and ADCC activation in our transmitting and nontransmitting dyads. First, we explored whether anti-UL16 IgG contributes to greater ADCC activation using statistical modeling. Our interaction analysis demonstrated that the association between maternal sera ADCC activation and reduced risk of cCMV transmission was stronger in dyads with higher UL16-specific IgG binding (Table 2). These data indicate that anti-UL16 IgG enhances protective ADCC responses in nontransmitting pregnancies. Next, we visualized the correlations between anti-UL16 IgG binding and anti-viral ADCC stratified by cCMV status. These scatterplots show that UL16-specific IgG responses and ADCC were strongly correlated and higher magnitude in nontransmitting dyads (Figure 6, A and B). Lastly, we measured NK cell degranulation against fibroblasts transduced with a recombinant adenovirus (rAd) vector expressing UL16 to quantify UL16-specific ADCC. We found that maternal sera from nontransmitting dyads with high UL16-specific IgG binding to FcγRIII stimulated potent ADCC against UL16-expressing fibroblasts (Figure 6, C and D). Taken together, our data support a model wherein anti-UL16 IgG binding to FcγRIII/CD16 on NK cells may mediate protective ADCC responses against HCMV transmission in utero (Figure 6E) — a hypothetical mechanism of protection that should be tested in future prospective clinical cohorts and experimental studies.

## Discussion

Our finding that ADCC-activating antibodies are associated with reduced risk of HCMV transmission in utero contributes to accumulating evidence that Fc effector functions should be explored in HCMV correlates and vaccine studies (7, 12, 13). During coevolution with the human immune system, HCMV has developed numerous strategies to evade Fc-mediated immunity including ADCC (15). HCMV encodes multiple viral FcγR decoys that bind to host IgG to prevent FcγR engagement (16, 17, 20) and employs at least 12 viral proteins to subvert NK cell killing by engaging inhibitory receptors, removing ligands for activating receptors, and interfering with immunological synapse formation (14, 15, 18, 24, 25). Vlahava et al. recently demonstrated that monoclonal antibodies and pooled polyclonal sera targeting the NK cell immunoevasins UL141 and UL16 can activate ADCC (21), yet our study is the first to our knowledge to demonstrate that IgG binding against these antigens correlates with FcγRIII activation and NK cell degranulation in a large mother-infant clinical cohort (*n* = 162 samples). Interestingly, anti-UL16, but not anti-UL141, IgG responses were associated with reduced risk of cCMV transmission in our study. Moreover, both the quantity and quality, assessed by FcγRIII engagement and ADCC activation, of anti-UL16 IgG was greater in nontransmitting dyads. Since ADCC activation and anti-UL16 IgG levels were lower in dyads with low/intermediate avidity scores, these responses may take substantial time to develop after infection, which may partially contribute to the association with reduced transmission risk since women with primary infection have higher cCMV transmission rates that those with nonprimary infection. These data lead us to speculate that ADCC-activating and UL16-specific antibodies could be primed by HCMV vaccination prior to pregnancy. UL16 blocks ligand interactions with the host NK cell activating receptor NKG2D and is highly conserved across clinical HCMV strains (26–28). Thus, targeting UL16 to overcome NK cell immune evasion strategies and activate maternal ADCC may be a promising strategy to prevent HCMV transmission in utero.

Our study has broad implications for HCMV vaccinology and immunotherapeutic development beyond congenital infection. Neutralizing antibodies against envelope glycoproteins has been a main focus of HCMV vaccines and antibody-based therapeutics (4, 29), yet there is an increasing appreciation in the field that nonneutralizing functions — i.e., antibody-mediated activation of cellular immunity — are also a key component of host defense against HCMV. Several studies have demonstrated that protection from the gB/MF59 subunit vaccine, which achieved 50% efficacy in preventing HCMV acquisition in clinical trials, was mediated by nonneutralizing antibody functions, likely against the cell surface conformation of gB (12, 13, 30, 31). Antibodies elicited by the gB/MF59 vaccine stimulated robust monocyte phagocytosis but not NK cell degranulation (12, 13), suggesting that these vaccine-induced antibodies activate ADCP but not ADCC. Although neutralization was poorly elicited in the gB/MF59 trial, anti-gB antibodies generated in natural infection likely mediate both neutralizing and nonneutralizing functions. Such polyfunctional antibodies have been observed in HIV infection (32–34), suggesting that anti-gB IgG may similarly protect against HCMV through multiple mechanisms. Nevertheless, anti-gB IgG responses were not associated with reduced risk of cCMV transmission in our cohort. These disparate results and our previous work (7) lead us to speculate that anti-gB antibodies may be more important for protection against initial virus acquisition, as was tested in the gB/MF59 trial, rather than HCMV transmission in utero. Moreover, vaccine-elicited anti-gB antibodies may mediate protection differently than in natural infection. In our study, antibodies against tegument or nonstructural proteins, and not entry envelope glycoproteins proteins, were correlated with decreased transmission risk and had better engagement of FcγRIII (21, 35). In particular, we found that maternal IgG elicited in natural infection against immunoevasin UL16 can effectively engage both low- and high-affinity FcγRIII and activate ADCC. Taken together with prior findings, these results highlight that both nonstructural (e.g., UL16) and structural (e.g., gB) HCMV antigens may need to be targeted to stimulate robust ADCC and ADCP, respectively. Overall, our work reinforces that diverse antigens and Fc antibody effector functions should be explored as immunologic targets against HCMV.

Further studies are needed to understand why certain antibodies against HCMV may engage FcγRs and activate downstream Fc effector functions better than others. Fc-mediated antibody responses are influenced by IgG Fc region characteristics such as IgG subclass and glycosylation that modify FcγR binding affinity (36–41). Our finding that the Fc region of HCMV-specific IgG in nontransmitting dyads had better-quality FcγRIII binding and activation suggests that there are differences in the Fc profiles of transmitting versus nontransmitting pregnancies. Fc region modifications to improve anti-UL16 and anti-UL141 IgG binding to FcγRIII can enhance ADCC and NK cell killing of HCMV-infected cells in vitro (21). Thus, Fc-engineering could be employed in the future to improve upon antibody-based therapeutics to prevent cCMV infection (36). Modulating IgG Fc region characteristics to augment Fc engagement could improve passive immunization strategies, which are needed, given the lack of efficacy of HCMV-HIG in randomized clinical trials (8, 9). It is interesting to speculate whether ineffective engagement of Fc-mediated immunity may have partially contributed to the failure of HCMV-HIG to prevent fetal transmission in prior clinical trials. We previously observed that FcγRI-mediated ADCP of HCMV was greatly reduced in the setting of high HCMV-HIG concentrations (7), leading us to hypothesize that certain Fc effector functions may have been poorly elicited in pregnant people treated with HCMV-HIG. Overall, our work highlights that Fc characteristics should be considered when designing next-generation polyclonal or monoclonal antibodies against HCMV. Moreover, novel vaccine strategies such as adjuvants to elicit specific IgG subclasses or glycosylation profiles endogenously to enhance ADCC or ADCP should also be explored.

The development of antibody-based prophylaxis and/or vaccines to prevent cCMV transmission has been hindered by our incomplete understanding of protective maternal immunity. Whether antibodies mediate protection solely by limiting systemic maternal viral replication and placental infection or also play a role in the fetal circulation remains unclear. We found that HCMV-specific ADCC-activating antibodies were poorly transferred from maternal to cord blood sera in our cohort, regardless of transmission status. In contrast, ADCC-mediating antibodies against influenza and pertussis have been shown to be robustly transferred across the placenta in healthy pregnancies (40). Since Fc region characteristics also govern transplacental IgG transport, Fc profiles likely underlie these differences in placental IgG transfer (38, 40, 41). Nevertheless, cord blood sera ADCC responses were strongly associated with protection, and anti-UL16 IgG was highly transferred from maternal to cord blood sera of uninfected infants. Vaaben et al. recently observed that cord blood NK cells expressing FcγRIII/CD16 are expanded in utero following cCMV infection (42, 43). Taken together, these studies suggest that maternal ADCC-activating antibodies transferred across the placenta and fetal NK cells expressing FcγRIII may synergize to defend against HCMV (42, 43). Therefore, strategies to enhance placental transfer of these potentially protective antibodies through Fc-engineering and to engage fetal and/or neonatal innate immune cells in Fc-mediated immunity should be explored.

Our study is limited by its retrospective design and relatively small sample size that reduced statistical power. Due to the cross-sectional nature of this cord blood bank donor cohort, we could not identify the timing of maternal HCMV acquisition or transmission during pregnancy. Thus, caution is warranted in interpreting our results since differences may be biased by a higher rate of primary infection, reinfection, and/or reactivation in transmitting versus nontransmitting dyads. This limitation and our sensitivity analyses excluding dyads with low/intermediate avidity scores or HCMV-specific IgM highlights the need for future longitudinal prospective studies to investigate protective immunity across gestation in maternal primary and nonprimary infection. Since all cord blood donors in the study were born healthy and longitudinal clinical data were not collected retrospectively, we could not assess clinical correlations between antibody responses and symptomatic versus asymptomatic infection. Notably, the vast majority of cCMV infections are asymptomatic at birth, yet many of these children will go on to develop delayed sequelae such as sensorineural hearing loss and neurodevelopmental problems (1, 44–47). Therefore, defining immune correlates of protection against cCMV transmission remains an important clinical endpoint for understanding protective maternal immunity and HCMV vaccine development (11). Maternal PBMCs were not collected, so we were also unable to assess cellular immunity. Whether maternal NK cell abundance, phenotype, or function differs in transmitting and nontransmitting pregnancies should be investigated. Since sera sample volumes were limited, we were only able to measure UL16-specific ADCC in a small subset of samples, and additional experimental work is needed to define the anti-viral functions of ADCC/FcγRIII activation and anti-UL16 IgG in controlling viral replication.

In conclusion, our study indicates that ADCC/FcγRIII activating antibodies and IgG directed against the NK cell immunoevasion protein UL16 may help protect against cCMV transmission. Our work suggests that designing HCMV vaccines or antibody-based therapeutics that can engage FcγRs and overcome NK cell immune evasion strategies may be an effective approach to combating this ubiquitous herpesvirus that is the leading infectious cause of congenital disease and disability worldwide.

## Methods

### Study population.

We analyzed maternal (*n* = 81) and cord blood (*n* = 81) sera samples from a retrospective cohort of mother-infant donors to the Carolinas Cord Blood Bank (CCBB), which has been previously described in our recent complementary study (Supplemental Figure 1) (7). All mothers in our study were HCMV IgG seropositive, and cases of cCMV infection were identified by HCMV DNAemia, detected by PCR, in the cord blood plasma at birth. “HCMV transmitting” cases with cCMV infection (*n* = 41) were matched to a target of 1 “HCMV nontransmitting” mother-infant dyad (*n* = 40). Maternal HCMV IgG seropositivity was confirmed by a whole-virion HCMV ELISA, and HCMV IgM seropositivity was determined using a clinical diagnostic ELISA (Bio-Rad CMV IgM EIA Kit). HCMV-specific IgG RAI scores were determined by calculating the mean RAI across 3 HCMV trains (TB40/E, AD169r, and Toledo virus) using whole-virion ELISA with urea as the dissociation agent as previously described (7). Maternal RAI scores < 60% were defined as low/intermediate avidity, and RAI scores ≥ to 60% were defined as high avidity (22). Matching criteria included infant sex, infant race, maternal age, and delivery year. Only people with healthy, uncomplicated pregnancies who gave birth at term were included in our study; cord blood donors were screened for signs of (a) neonatal sepsis, (b) congenital infection (petechial rash, thrombocytopenia, hepatosplenomegaly), and (c) congenital abnormalities.

### NK cell–mediated ADCC.

NK cell degranulation was quantified by cell-surface expression of CD107a as previously described (48). MRC-5 fibroblasts (target cells; ATCC) were infected with HCMV strain AD169r (a AD169 derivative with repaired UL128-131 expression named BadrUL131-Y4-GFP; ref. 49) at an MOI of 1.0 or mock infected. After 48 hours, primary human NK cells (effector cells) were isolated by negative selection with magnetic beads (human NK cell isolation kit; Miltenyi Biotec) from PBMCs of a healthy adult donor; then, live, primary NK cells were added to each well containing HCMV-infected or mock-infected fibroblasts at an effector/target (E:T) ratio of 1:1. Cytogam IgG product or diluted sera samples (1:75) were then added with brefeldin A (GolgiPlug; BD Biosciences), monensin (GolgiStop; BD Biosciences), and anti–CD107a FITC (clone H4A3; BD Biosciences). After a 6-hour incubation, NK cells were stained with anti–CD56-PE/Cy7 (clone NCAM16.2; BD Biosciences), anti–CD16-PacBlue (clone 3G8; BD Biosciences), and a viability dye (Live/Dead Aqua Dead Cell Stain, Thermo Fisher Scientific). Events were acquired on an LSR Fortessa flow cytometer, and the frequencies of live, CD107a^+^ NK cells were calculated in FlowJo (gating strategy in Supplemental Figure 2). To correct for nonspecific degranulation activity, the signal in mock-infected wells was subtracted from the signal in HCMV-infected wells for each sera sample (Supplemental Figure 2). To measure HCMV UL16–specific ADCC, the same assay approach was used to measure NK cell degranulation against immortalized human fetal foreskin fibroblasts expressing the coxsackie adenovirus receptor (HFFF-hCARs) (50) that were transfected with rAd encoding HCMV UL16 (21, 51). To control for nonspecific degranulation, parallel assays were performed with control rAD transfected HFFF-hCARs, and the NK cell degranulation activity against both targets is reported. Maternal sera in the UL16 ADCC assay was tested at a 1:10 and 1:50 dilution.

### HCMV antigen-specific IgG binding.

IgG binding to HCMV antigens including UL16 and UL141 (in-house), envelope glycoproteins (gB ectodomain, pentamer complex, gH/gL/gO, gH/gL), and tegument proteins (pp28, pp150, pp52) was quantified using a BAMA as previously described (7). In brief, HCMV antigens were coupled to intrinsically fluorescent beads (Bio-Plex pro magnetic COOH beads, Bio-Rad) and were then coincubated with serially diluted Cytogam IgG product (CSL Behring) or sera samples. Antigen-specific IgG binding was detected with mouse anti–human IgG-PE (Southern Biotech), and MFI was acquired on a Bio-Plex 200.

### HCMV antigen-specific IgG binding to FcγRs.

Antigen-specific IgG binding to FcγRIII/CD16 was measured using a modified BAMA as previously described (7). Purified human FcγRIII high-affinity (V158) and low-affinity (F158) variants were produced by the DHVI Protein Production Facility and biotinylated in-house. First, sera samples were coincubated with HCMV antigen–coated beads, as above. Next, biotinylated FcγRIII was complexed with streptavidin-PE (BD Biosciences) and was then coincubated with antibody-bound beads. MFI was acquired on a Bio-Plex 200.

### FcγR IgG activation.

HCMV-specific IgG activation of FcγRIII/CD16 was quantified using mouse BW thymoma cells expressing chimeric FcγR-CD3ζ as previously described (7, 52). To confirm FcγR expression, BW cells were stained with anti–FcγRI/CD64-PE (clone 10.1, eBioscience), anti–FcγRII/CD32-PE (clone 6C4, eBioscience), anti–FcγRIII/CD16-PE (clone CB16, eBioscience), and anti–Ig-PE isotype control (clone P3.6.2.8.1, eBioscience). Events were acquired on a LSRII flow cytometer and were then analyzed using FlowJo. To quantify FcγR activation, 96-well plates were coated with HCMV strain AD169r (20,000 PFU/well) or UL16 antigen (250 μg/well) and were then coincubated with Cytogam IgG product or sera samples (diluted 1:10) to form immune complexes. Next, FcγRIII-expressing BW cells were added and incubated for 20 hours. Cell supernatants were then harvested, and mouse IL-2 levels were quantified as a read-out of FcγRIII-CD3ζ activation using ELISA as previously described (7).

### Statistics.

All primary data underwent independent quality control by another lab member using standardized criteria based on duplicate well variance and performance of positive and negative controls, which included Cytogam, HCMV seropositive, and HCMV seronegative sera samples. Wilcoxon rank-sum tests were used to compare transmitting and nontransmitting dyads, and Wilcoxon signed-rank tests were used to assess differences within dyads. Spearman’s correlation coefficient was calculated for select immune variables, and correlation matrices were plotted using the corrplot package in R v4.1. ELISA. BAMA data were log-transformed for all regression analyses. Statistical significance was defined a priori as *P* < 0.05 after an FDR correction for multiple comparisons. Statistical analyses were completed in R v4.1 and GraphPad Prism v9.1.

### Study approval.

Approval was obtained from Duke University School of Medicine’s IRB (no. Pro00089256) to use deidentified clinical data and biospecimens provided by the CCBB. No patients were prospectively recruited for this study, and all samples were acquired retrospectively from the CCBB biorepository from donors who had previously provided written consent for banked biospecimens to be used for research.

### Data availability.

All individual data points included in the figures and statistical analyses are available in the supplemental data provided. Additional supporting data are available in the supplemental materials and from the corresponding author upon request.

## Author contributions

ECS, JP, and SRP designed the research study. ECS, IGM, NR, and CTP conducted the experiments and acquired the data. ECS, IGM, and NR completed the primary data analysis. ECS completed the statistical analyses with oversight from KMW. SRP and KMW acquired funding for the study. JHH helped acquire the human samples. RJS provided key reagents for the study. ECS wrote the primary draft of the manuscript. ECS, IGM, NR, CTP, JHH, KMW, JP, RJS, and SRP contributed to writing and editing the manuscript.

## Supplementary Material

Supplemental data

Supporting data values

## Figures and Tables

**Table 2:**
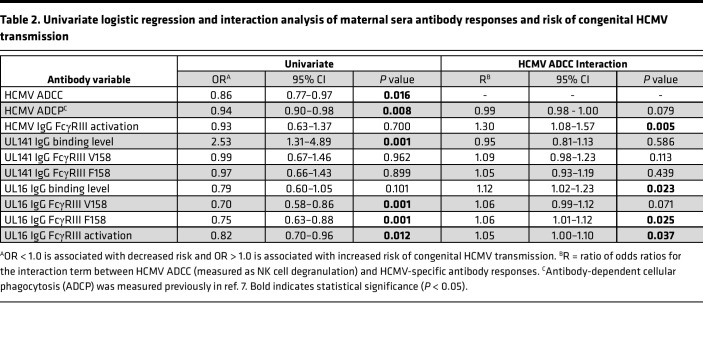
Univariate logistic regression and interaction analysis of maternal sera antibody responses and risk of congenital HCMV transmission

**Table 1:**
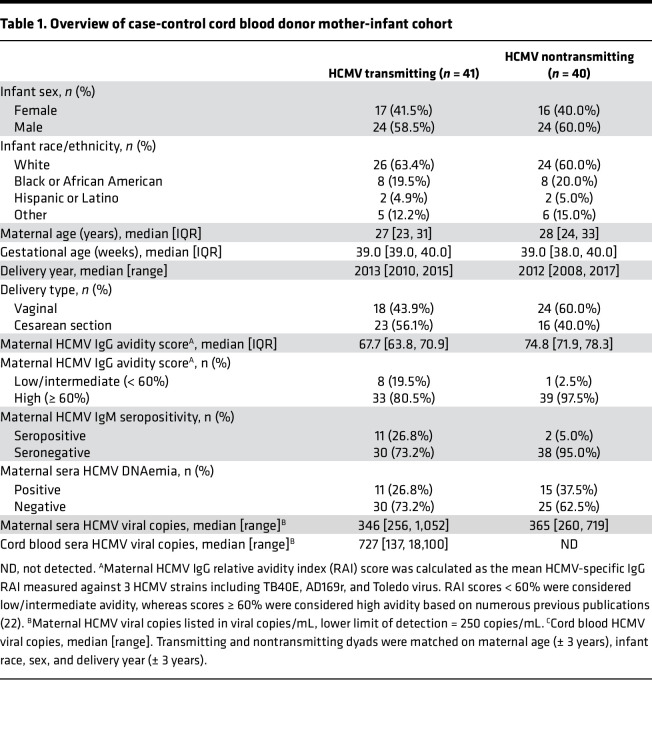
Overview of case-control cord blood donor mother-infant cohort

**Table 3:**
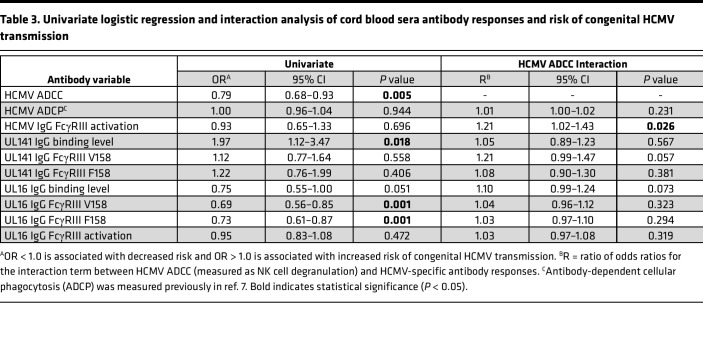
Univariate logistic regression and interaction analysis of cord blood sera antibody responses and risk of congenital HCMV transmission

## Version 1 07/10/2023

Electronic publication
