# Supplementary material for: ADCC-activating antibodies correlate with decreased risk of congenital human cytomegalovirus transmission
Source: JCI Insight. 2023 Jul 10;8(13):e167768. doi: 10.1172/jci.insight.167768 (PMC10371338; doi:10.1172/jci.insight.167768)
Supplement: Supplemental data [file jciinsight-8-167768-s040.pdf]

Supplementary Figure 1. Identification of HCMV transmitting and non-transmitting mother-infant dyads from the Carolinas Cord Blood Bank (CCBB) biorepository.

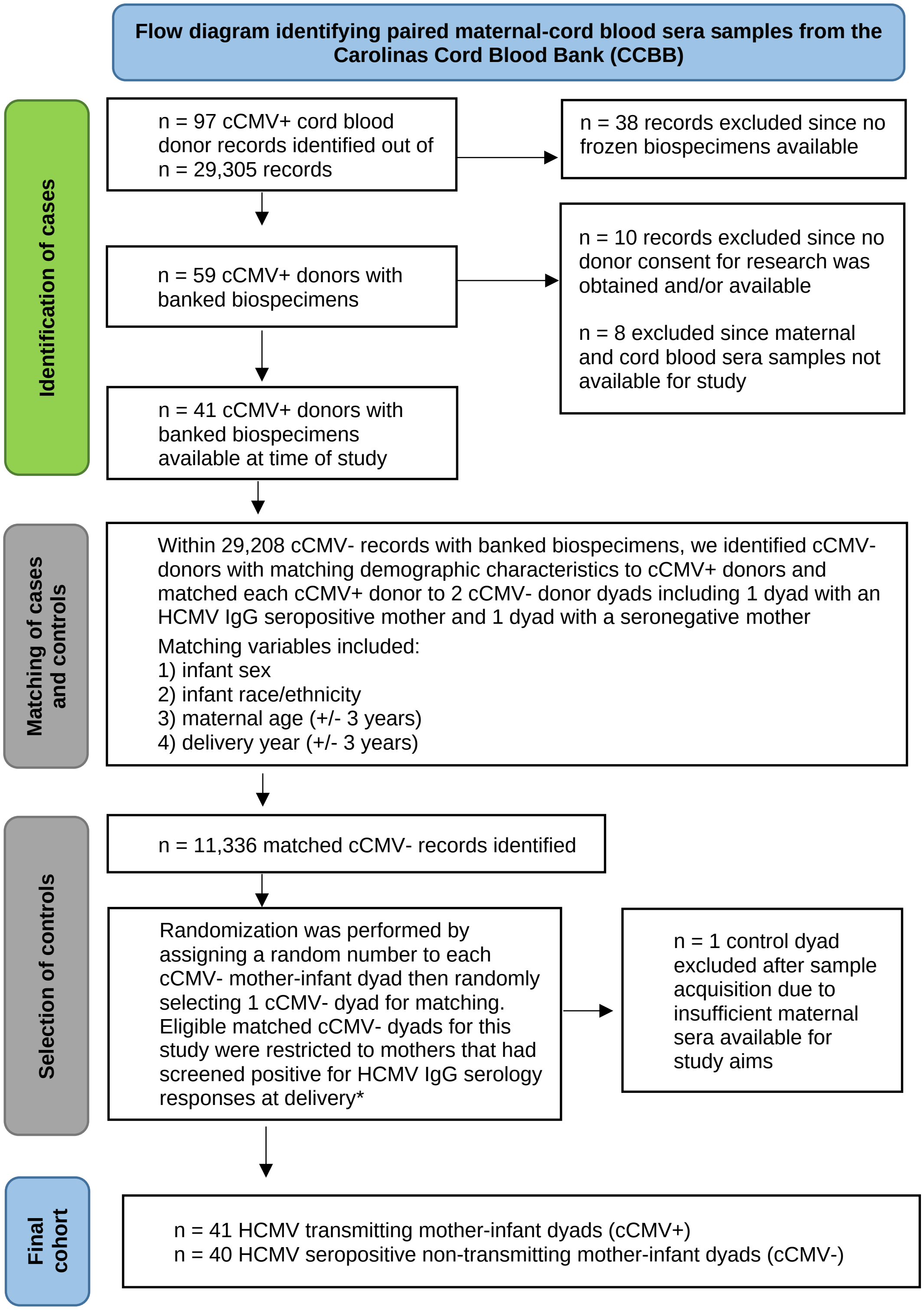

cCMV = congenital HCMV infection
cCMV+ = positive HCMV PCR cord blood screening at birth
cCMV- = negative HCMV PCR cord blood screening at birth

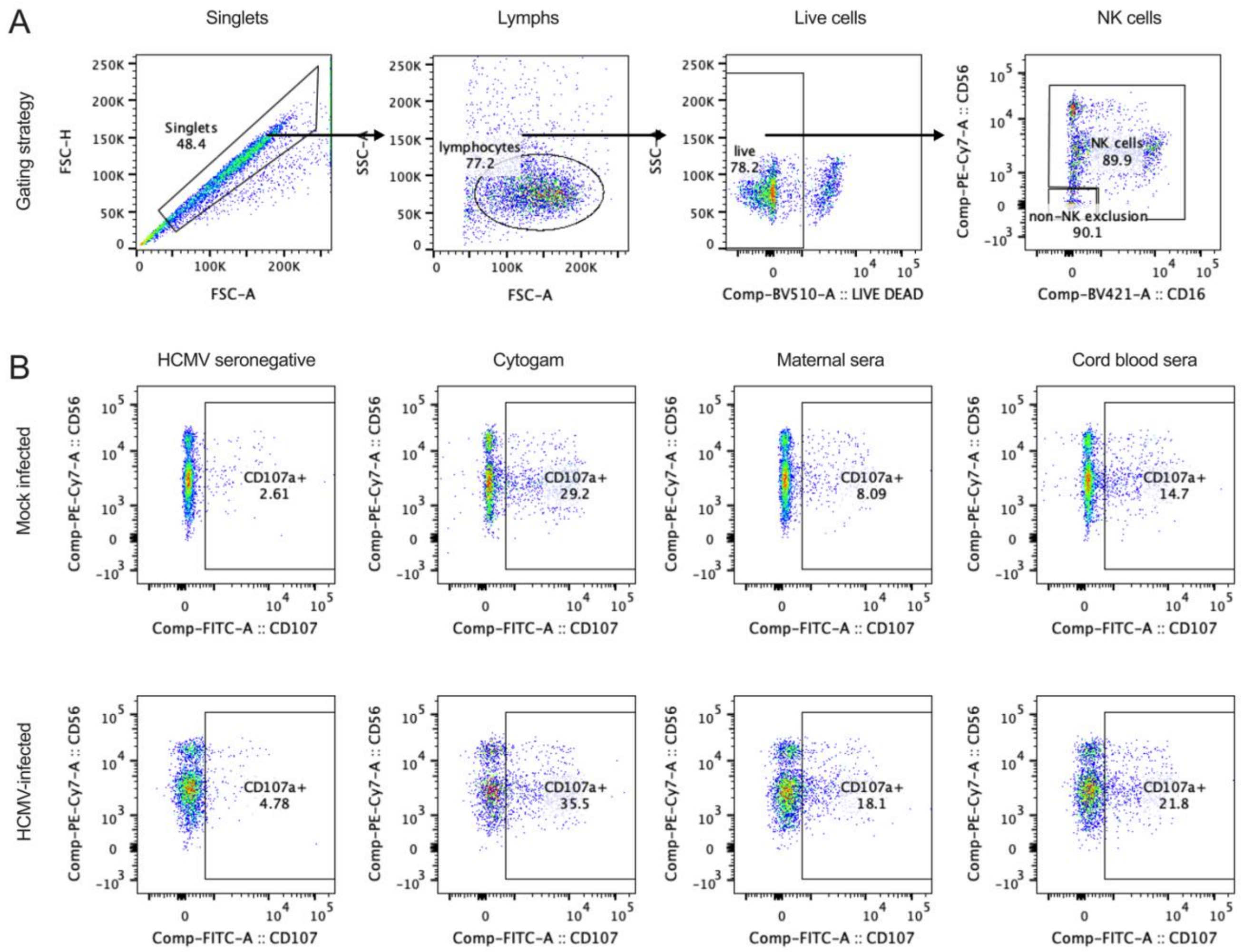

Supplementary Figure 2. NK cell degranulation gating strategy for ADCC assay. Antibody-dependent cellular cytotoxicity (ADCC) was measured by quantifying NK cell degranulation (i.e., CD107a positivity) against HCMV-infected fibroblasts. PBMCs were rested overnight then primary NK cells were isolated by negative selection with magnetic beads prior to co-incubation with HCMV-infected and mock-infected cells. (A) Gating strategy: singlets → lymphocytes → live lymphocytes → NK cells → CD107a positive cells. (B) CD107a positive cells were identified in the NK cell gate under mock-infected and HCMV-infected (MOI = 1.0) conditions for HCMV seronegative, Cytogam, and HCMV seropositive maternal and cord blood sera samples. HCMV-specific ADCC activating antibody responses were calculated by subtracting the % CD107a positive cells in the mock-infected condition from the % CD107a positive cells in the HCMV-infected condition for each sample.

Supplementary Table 1. Maternal sera HCMV-specific antibody responses in HCMV transmitting versus non-transmitting dyads

	HCMV transmitting (n = 41)		HCMV non-transmitting (n = 40)			
**Variable**	**Median**	**IQR**	**Median**	**IQR**	**p value**	**FDR p value**
HCMV ADCC	7.5	5.7	9.6	6.0	**0.012**	**0.020**
HCMV IgG FcγRIII activation	9481.4	7494.3	12864.4	13557.8	0.054	0.076
UL16 IgG binding level	32.3	92.6	99.8	342.8	**0.019**	**0.029**
UL16 IgG FcγRIII V158	13.5	53.5	420.6	1028.1	**<0.001**	**<0.001**
UL16 IgG FcγRIII F158	1.0	0.0	119.4	391.2	**<0.001**	**<0.001**
UL16 IgG FcγRIII activation	0.3	2.8	5.2	61.3	**0.002**	**0.005**
UL141 IgG binding level	465.0	460.0	341.9	351.7	**0.010**	**0.017**
UL141 IgG FcγRIII V158	833.0	480.3	736.7	770.4	0.360	0.440
UL141 IgG FcγRIII F158	431.5	439.5	484.9	411.1	0.640	0.690
gB-transfected cell IgG binding	24.4	2.7	21.1	7.1	**0.005**	**0.009**
gB IgG binding level	6917.5	9747.1	1591.3	3126.6	**<0.001**	**0.001**
gB IgG FcγRIII V158	798.5	2605.7	418.3	709.8	**0.049**	0.070
gB IgG FcγRIII F158	181.5	1237.2	150.4	253.8	0.290	0.370
pentamer IgG binding level	7788.8	12098.0	1471.8	2600.2	**<0.001**	**<0.001**
pentamer FcγRIII V158	3506.5	5463.3	854.6	1138.3	**<0.001**	**<0.001**
pentamer IgG FcγRIII F158	1746.0	4114.3	391.9	450.5	**<0.001**	**<0.001**
gHgLgO IgG binding level	9468.0	14464.4	1666.9	3341.4	**<0.001**	**<0.001**
gHgLgO FcγRIII V158	3235.3	7297.0	630.3	976.7	**<0.001**	**<0.001**
gHgLgO IgG FcγRIII F158	1598.0	4973.8	270.7	612.2	**<0.001**	**<0.001**
gHgL IgG binding level	704.8	893.2	213.2	296.2	**<0.001**	**<0.001**
gHgL FcγRIII V158	102.0	270.5	51.4	64.2	**0.009**	**0.016**
gHgL IgG FcγRIII F158	22.0	106.0	10.9	19.5	0.094	0.130
pp52 IgG binding level	6774.3	9980.0	1077.5	2805.6	**<0.001**	**<0.001**
pp52 FcγRIII V158	6120.0	10500.3	1982.3	4796.3	**0.003**	**0.006**
pp52 IgG FcγRIII F158	3839.3	8998.2	1351.2	2793.8	**0.011**	**0.018**
pp28 IgG binding level	2606.8	5139.5	1689.3	3689.4	0.071	0.097
pp28 FcγRIII V158	2503.5	4755.5	2454.5	3950.9	0.980	0.980
pp28 IgG FcγRIII F158	557.5	3223.8	934.2	2410.5	0.390	0.460
pp150 IgG binding level	16009.3	17044.8	7059.9	14579.7	**0.006**	**0.011**
pp150 FcγRIII V158	10488.3	10381.3	8811.8	8544.8	0.360	0.440
pp150 IgG FcγRIII F158	9012.8	14228.7	7410.2	9211.9	0.500	0.560

Bold indicates statistical significance ($p < 0.05$).

Supplementary Table 2. Cord blood sera HCMV-specific antibody responses in HCMV transmitting versus non-transmitting dyads						
	HCMV transmitting (n = 41)		HCMV non-transmitting (n = 40)			
**Variable**	**Median**	**IQR**	**Median**	**IQR**	**p value**	**FDR p value**
HCMV ADCC	4.8	3.8	7.1	4.0	**0.001**	**0.003**
HCMV IgG FcγRIII activation	8790.8	7983.3	12847.7	14424.3	**0.008**	**0.015**
UL16 IgG binding level	19.0	54.4	125.4	278.5	**0.002**	**0.004**
UL16 IgG FcγRIII V158	11.0	37.3	284.3	754.9	**<0.001**	**<0.001**
UL16 IgG FcγRIII F158	1.0	4.3	70.8	265.2	**<0.001**	**<0.001**
UL16 IgG FcγRIII activation	0.3	6.5	1.4	21.0	0.400	0.470
UL141 IgG binding level	385.5	361.2	302.0	352.9	**0.035**	0.051
UL141 IgG FcγRIII V158	783.5	644.5	677.3	771.5	0.420	0.490
UL141 IgG FcγRIII F158	476.3	354.0	496.3	446.6	0.970	0.980
gB-transfected cell IgG binding	24.0	2.1	21.8	6.7	**0.002**	**0.003**
gB IgG binding level	4733.0	7638.2	1824.2	1945.2	**<0.001**	**0.001**
gB IgG FcγRIII V158	750.8	1714.5	305.1	441.3	**0.016**	**0.026**
gB IgG FcγRIII F158	163.8	446.7	95.9	156.6	0.200	0.260
pentamer IgG binding level	10772.1	16600.4	952.1	1904.4	**<0.001**	**<0.001**
pentamer FcγRIII V158	6350.3	7475.2	606.6	751.5	**<0.001**	**<0.001**
pentamer IgG FcγRIII F158	3059.0	5823.8	281.7	335.8	**<0.001**	**<0.001**
gHgLgO IgG binding level	10785.4	18236.0	973.3	2280.3	**<0.001**	**<0.001**
gHgLgO FcγRIII V158	6267.8	10928.2	424.9	741.7	**<0.001**	**<0.001**
gHgLgO IgG FcγRIII F158	3482.8	6573.0	138.0	218.3	**<0.001**	**<0.001**
gHgL IgG binding level	772.3	1562.0	123.6	225.3	**<0.001**	**<0.001**
gHgL FcγRIII V158	122.3	313.2	37.2	49.5	**0.001**	**0.002**
gHgL IgG FcγRIII F158	20.0	91.8	8.7	12.9	**0.017**	**0.027**
pp52 IgG binding level	6894.0	7742.3	634.3	2465.6	**<0.001**	**<0.001**
pp52 FcγRIII V158	6641.0	7955.7	1486.8	4117.8	**<0.001**	**<0.001**
pp52 IgG FcγRIII F158	3809.0	8143.0	922.7	2610.6	**0.001**	**0.002**
pp28 IgG binding level	1459.0	1972.8	1383.7	2528.4	0.310	0.390
pp28 FcγRIII V158	1226.5	2212.0	1623.0	3973.5	0.880	0.910
pp28 IgG FcγRIII F158	275.0	989.2	697.9	2428.9	0.370	0.440
pp150 IgG binding level	20199.0	14946.0	6307.0	15201.4	**0.001**	**0.003**
pp150 FcγRIII V158	13487.3	8827.5	7418.3	11574.3	**0.020**	**0.030**
pp150 IgG FcγRIII F158	11753.8	12327.8	5710.8	10773.8	**0.029**	**0.042**
Bold indicates statistical significance ($p < 0.05$).						

Supplementary Table 3. Maternal sera antibody responses in dyads with low/intermediate versus high HCMV IgG avidity scores [a]

	Low/intermediate RAI score (n = 9)		High RAI score (n = 72)			
**Variable**	**Median**	**IQR**	**Median**	**IQR**	**p value**	**FDR p value**
HCMV ADCC	5.1	2.6	9.5	5.8	**0.001**	**0.004**
HCMV IgG FcγRIII activation	7041.7	1067.1	12517.9	11502.6	**<0.001**	**0.002**
UL16 IgG binding level	20.2	13.2	90.9	259.2	**0.002**	**0.008**
UL16 IgG FcγRIII V158	1.9	9.2	90.3	949.4	**<0.001**	**0.001**
UL16 IgG FcγRIII F158	1.0	0.0	11.8	389.1	**0.003**	**0.011**
UL16 IgG FcγRIII activation	0.3	0.0	2.6	35.2	**0.022**	**0.041**
UL141 IgG binding level	296.5	239.3	419.5	330.1	0.130	0.170
UL141 IgG FcγRIII V158	507.0	498.5	850.8	772.9	**0.004**	**0.012**
UL141 IgG FcγRIII F158	189.3	186.9	488.3	471.5	**<0.001**	**0.001**
gB-transfected cell IgG binding	23.0	4.5	23.8	4.8	0.550	0.600
gB IgG binding level	2740.6	3899.8	3637.0	7335.6	0.470	0.530
gB IgG FcγRIII V158	292.4	419.9	651.0	1625.3	0.100	0.140
gB IgG FcγRIII F158	42.7	137.1	191.8	636.5	**0.041**	0.065
pentamer IgG binding level	5601.5	8726.1	3709.0	7396.3	0.550	0.600
pentamer FcγRIII V158	1999.8	2860.0	1896.0	3028.6	0.840	0.870
pentamer IgG FcγRIII F158	762.3	1864.7	651.8	1922.8	0.790	0.830
gHgLgO IgG binding level	4950.7	10157.5	4640.0	8556.6	0.960	0.960
gHgLgO FcγRIII V158	1369.5	3219.3	1750.0	3651.3	0.900	0.920
gHgLgO IgG FcγRIII F158	313.7	1349.5	716.0	2401.6	0.570	0.610
gHgL IgG binding level	249.2	330.3	447.5	689.6	0.200	0.250
gHgL FcγRIII V158	33.9	109.5	68.8	110.6	0.140	0.180
gHgL IgG FcγRIII F158	2.8	22.6	14.8	42.8	0.070	0.100
pp52 IgG binding level	10843.7	13307.5	2494.5	5583.1	**0.010**	**0.022**
pp52 FcγRIII V158	6891.3	12057.0	3681.0	6418.1	0.140	0.180
pp52 IgG FcγRIII F158	4038.3	8863.0	1874.8	4564.4	0.240	0.300
pp28 IgG binding level	1787.3	1468.2	2465.3	4081.5	0.280	0.340
pp28 FcγRIII V158	1079.7	1475.8	2537.3	5050.5	**0.028**	**0.049**
pp28 IgG FcγRIII F158	220.3	349.1	888.3	3529.0	**0.008**	**0.019**
pp150 IgG binding level	11497.6	17510.0	10391.3	18133.3	0.950	0.960
pp150 FcγRIII V158	4789.9	8643.3	9824.5	9385.9	0.097	0.140
pp150 IgG FcγRIII F158	4026.3	6731.6	8581.0	12943.8	0.061	0.092

[a] HCMV IgG relative avidity index (RAI) score was calculated as the mean RAI measured against 3 HCMV strains. RAI scores <60% were considered low/intermediate avidity whereas scores ≥60% were considered high avidity. Bold indicates statistical significance ($p < 0.05$).

Supplementary Table 4. Maternal sera antibody responses in dyads with and without detectable HCMV-specific IgM [a]						
	HCMV IgM+ mothers (n=13)		HCMV IgM- mothers (n=68)			
**Variable**	**Median**	**IQR**	**Median**	**IQR**	**p value**	**FDR p value**
HCMV ADCC	6.8	8.0	9.1	5.7	0.130	0.240
HCMV IgG FcγRIII activation	7717.6	5689.2	12136.6	11592.6	**0.044**	0.120
UL16 IgG binding level	29.5	27.3	89.9	256.7	**0.026**	0.090
UL16 IgG FcγRIII V158	9.0	18.3	93.6	884.3	**0.001**	**0.017**
UL16 IgG FcγRIII F158	1.0	0.0	12.9	388.4	**0.002**	**0.022**
UL16 IgG FcγRIII activation	0.3	0.0	2.0	31.6	0.076	0.180
UL141 IgG binding level	376.3	355.7	417.2	339.7	0.860	0.920
UL141 IgG FcγRIII V158	669.3	506.5	838.2	780.6	0.084	0.180
UL141 IgG FcγRIII F158	296.8	286.5	484.9	498.4	**0.025**	0.090
gB-transfected cell IgG binding	22.9	4.7	23.8	4.8	0.900	0.960
gB IgG binding level	4245.5	11222.5	2762.0	5690.9	0.620	0.750
gB IgG FcγRIII V158	1262.0	1862.0	477.4	1047.3	0.460	0.610
gB IgG FcγRIII F158	445.0	692.7	158.1	364.7	0.590	0.730
pentamer IgG binding level	7703.5	15607.6	3711.1	6498.0	0.084	0.180
pentamer FcγRIII V158	3203.5	5246.5	1572.8	2766.1	0.073	0.180
pentamer IgG FcγRIII F158	2225.5	4176.2	611.8	1502.8	0.130	0.240
gHgLgO IgG binding level	9859.8	15935.0	4208.0	7848.2	0.077	0.180
gHgLgO FcγRIII V158	4511.0	5208.2	1235.8	2634.4	0.061	0.160
gHgLgO IgG FcγRIII F158	2445.5	4781.0	433.7	1354.2	0.091	0.190
gHgL IgG binding level	509.5	580.0	410.9	664.8	0.750	0.830
gHgL FcγRIII V158	63.8	266.5	69.2	98.2	0.550	0.700
gHgL IgG FcγRIII F158	7.0	63.3	15.1	32.5	0.340	0.470
pp52 IgG binding level	11638.8	14187.0	2429.8	5181.9	**0.003**	**0.023**
pp52 FcγRIII V158	7662.5	9836.8	3031.6	5465.0	**0.014**	0.063
pp52 IgG FcγRIII F158	4913.3	8810.5	1491.6	4591.1	**0.022**	0.085
pp28 IgG binding level	2606.8	5061.5	2209.0	3962.8	0.290	0.420
pp28 FcγRIII V158	1690.3	4247.3	2466.7	4595.6	0.990	0.990
pp28 IgG FcγRIII F158	555.3	2684.5	854.9	3196.8	0.650	0.780
pp150 IgG binding level	23035.8	11150.5	9472.6	17520.5	**0.016**	0.069
pp150 FcγRIII V158	14658.5	10975.8	9216.9	9331.6	0.190	0.310
pp150 IgG FcγRIII F158	14402.5	14151.7	7566.6	9674.8	0.240	0.360
Bold indicates statistical significance ($p < 0.05$).						

Supplementary Table 5. Univariate logistic regression analysis of antibody responses and risk of cCMV infection excluding HCMV IgG low/intermediate avidity mothers

Antibody variable	Maternal sera			Cord blood sera		
	**OR[a]**	**95% CI**	**p value**	**OR**	**95% CI**	**p value**
**HCMV ADCC**	0.89	0.78 - 1.01	0.065	0.81	0.68 - 0.96	**0.017**
**HCMV IgG FcγRIII activation**	1.09	0.73 - 1.65	0.658	1.09	0.76 - 1.59	0.632
**UL16 IgG binding level**	0.87	0.65 - 1.16	0.341	0.83	0.62 - 1.12	0.228
**UL16 IgG FcγRIII V158**	0.76	0.61 - 0.94	**0.010**	0.70	0.56 - 0.88	**0.003**
**UL16 IgG FcγRIII F158**	0.79	0.67 - 0.94	**0.006**	0.75	0.62 - 0.90	**0.003**
**UL16 IgG FcγRIII activation**	0.85	0.73 - 1.00	**0.045**	1.00	0.87 - 1.15	0.993

[a] OR < 1.0 is associated with decreased risk and OR > 1.0 is associated with increased risk of congenital HCMV transmission. Bold indicates statistical significance ($p < 0.05$). n = 72 mother-infant dyads

Supplementary Table 6. Univariate logistic regression analysis of antibody responses and risk of cCMV infection excluding HCMV-specific IgM+ mothers

Antibody variable	Maternal sera			Cord blood sera		
	**OR[a]**	**95% CI**	**p value**	**OR**	**95% CI**	**p value**
**HCMV ADCC**	0.89	0.78 - 1.01	0.081	0.86	0.73 - 1.01	0.070
**HCMV IgG FcγRIII activation**	1.07	0.71 - 1.07	0.755	1.08	0.75 - 1.57	0.672
**UL16 IgG binding level**	0.92	0.68 - 1.23	0.572	0.87	0.64 - 1.18	0.386
**UL16 IgG FcγRIII V158**	0.78	0.63 - 0.96	**0.017**	0.72	0.58 - 0.90	**0.005**
**UL16 IgG FcγRIII F158**	0.80	0.68 - 0.95	**0.013**	0.76	0.63 - 0.92	**0.005**
**UL16 IgG FcγRIII activation**	0.86	0.73 - 1.01	0.068	1.01	0.87 - 1.17	0.895

[a] OR < 1.0 is associated with decreased risk and OR > 1.0 is associated with increased risk of congenital HCMV transmission. Bold indicates statistical significance ($p < 0.05$). n = 68 mother-infant dyads

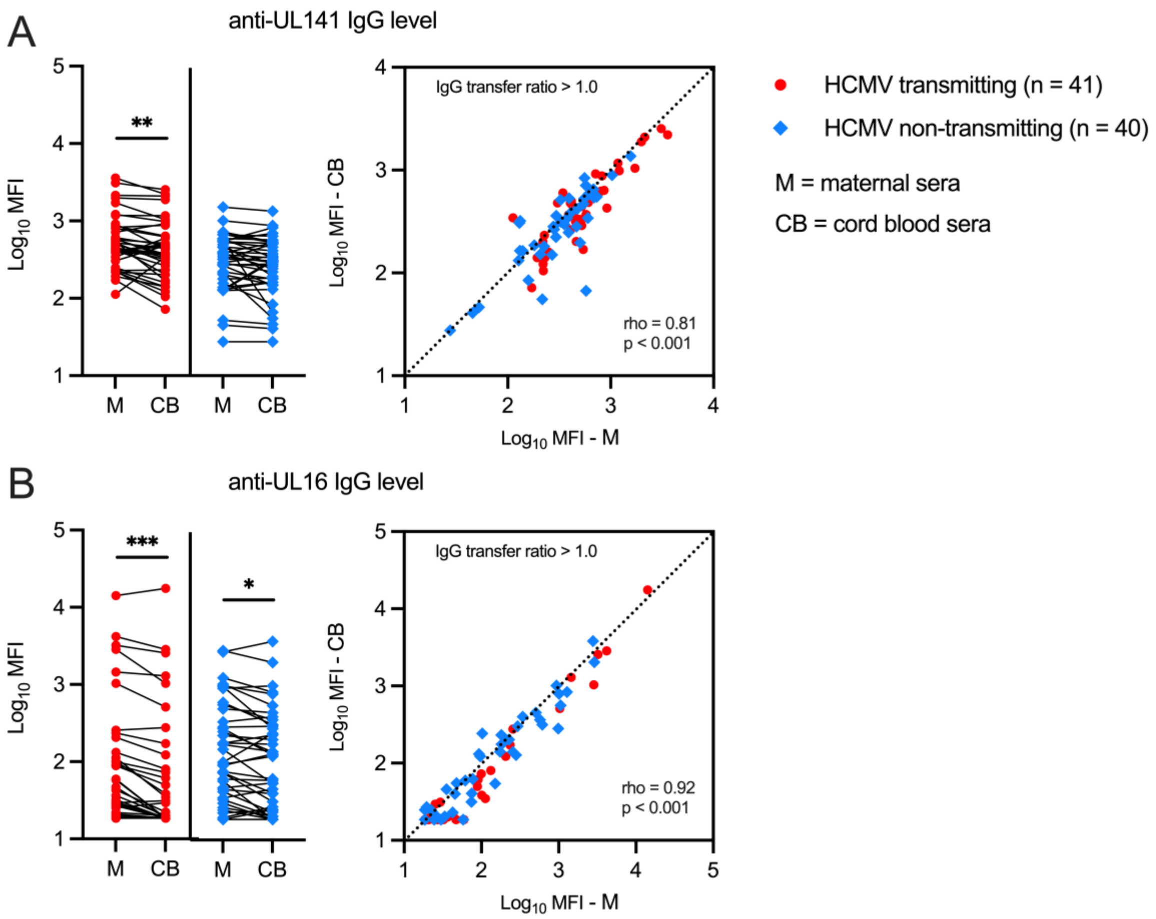

Supplementary Figure 3. Anti-UL141 and anti-UL16 IgG transfer from maternal to cord blood sera. Anti-UL141 and anti-UL16 IgG binding levels were measured with a binding level antibody multiplex assay using maternal (M) and cord blood (CB) sera from HCMV transmitting (red circles, n = 41) and non-transmitting (blue diamonds, n = 40) mother-infant dyads. (A) Anti-UL141 IgG level compared within mother-infant dyads and scatterplot showing Spearman correlation between anti-UL141 IgG level in paired maternal versus cord blood sera samples. (B) Anti-UL16 IgG level compared within mother-infant dyads and scatterplot showing Spearman correlation between anti-UL141 IgG level in paired maternal versus cord blood sera samples. FDR-corrected *P* values for Wilcoxon signed-rank test. * $P < 0.05$, ** $P < 0.01$, *** $P < 0.001$.
